# Targeting Mitochondrial Complex I Deficiency in MPP^+^/MPTP-induced Parkinson’s Disease Cell Culture and Mouse Models by Transducing Yeast *NDI1* Gene

**DOI:** 10.1186/s12575-024-00236-3

**Published:** 2024-04-09

**Authors:** Hongzhi Li, Jing Zhang, Yuqi Shen, Yifan Ye, Qingyou Jiang, Lan Chen, Bohao Sun, Zhuo Chen, Luxi Shen, Hezhi Fang, Jifeng Yang, Haihua Gu

**Affiliations:** 1 Key Laboratory of Laboratory Medicine, Ministry of Education, Wenzhou Key Laboratory of Cancer Pathogenesis and Translation, School of Laboratory Medicine and Life Sciences, Wenzhou Medical University, Chashan University Town, Northern Zhongshan Road, Wenzhou, 325035 China; 2 Department of Pathology, Second Affiliated Hospital, School of Medicine, Zhejiang University, Hangzhou, 310009 China; 3 Department of Internal Neurology, Beijing Friendship Hospital, Capital Medical University, Beijing, 100050 China

**Keywords:** Parkinson’s disease, MPTP, Respiratory chain complex, Yeast *NDI1* gene, Viral vector, Therapy

## Abstract

**Background:**

MPTP (1-methyl-4-phenyl-1,2,3,6-tetrahydropyridine), original found in synthetic heroin, causes Parkinson’s disease (PD) in human through its metabolite MPP^+^ by inhibiting complex I of mitochondrial respiratory chain in dopaminergic neurons. This study explored whether yeast internal NADH-quinone oxidoreductase (NDI1) has therapeutic effects in MPTP- induced PD models by functionally compensating for the impaired complex I. MPP^+^-treated SH-SY5Y cells and MPTP-treated mice were used as the PD cell culture and mouse models respectively. The recombinant *NDI1* lentivirus was transduced into SH-SY5Y cells, or the recombinant *NDI1* adeno-associated virus (rAAV5-*NDI1*) was injected into substantia nigra pars compacta (SNpc) of mice.

**Results:**

The study in vitro showed NDI1 prevented MPP^+^-induced change in cell morphology and decreased cell viability, mitochondrial coupling efficiency, complex I-dependent oxygen consumption, and mitochondria-derived ATP. The study in vivo revealed that rAAV-*NDI1* injection significantly improved the motor ability and exploration behavior of MPTP-induced PD mice. Accordingly, NDI1 notably improved dopaminergic neuron survival, reduced the inflammatory response, and significantly increased the dopamine content in striatum and complex I activity in substantia nigra.

**Conclusions:**

NDI1 compensates for the defective complex I in MPP^+^/MPTP-induced models, and vastly alleviates MPTP-induced toxic effect on dopaminergic neurons. Our study may provide a basis for gene therapy of sporadic PD with defective complex I caused by MPTP-like substance.

**Supplementary Information:**

The online version contains supplementary material available at 10.1186/s12575-024-00236-3.

## Introduction

The functional defects of mitochondrial respiratory chain NADH dehydrogenase complex (complex I) can cause a variety of diseases in nervous, muscular systems including Leber’s hereditary optic neuropathy, Leigh syndrome, Parkinson’s disease, etc [1, 2]. Among them, Parkinson’s disease (PD) is the second most common degenerative disease of nervous system, with clinical characteristics of resting tremor, bradykinesia, muscle rigidity, and abnormal posture and gait, affecting 1% and 3% of people over 60 and 80 years respectively [3]. The cytopathologic hallmark of PD is the progressive degeneration of dopaminergic neurons, and the pathogenesis may involve multiple etiologic factors such as genetics, environmental agents, and their interactions. Genetically, PD is grouped into familial and sporadic forms, of which about 95% are sporadic. Mitochondrial dysfunction due to the reduced activity of mitochondrial respiratory chain complex I has been implicated mainly in the pathogenesis of sporadic PD [4, 5]. Deficiencies in mitochondrial complex I can lead to the pathological features and clinical symptoms of PD [6–8]. This decreased complex I activity, which is prevalent in sporadic PD patients, is apparently not entirely due to mutations of some specific genes encoding subunits of complex I [9, 10]. Considering the complexity of these mutations, the more feasible therapeutic strategy should be to functionally compensate for the deficiency of the entire complex I, not to correct various specific mutations.

MPTP (1-methyl-4-phenyl-1,2,3,6-tetrahydropyridine) found in synthetic heroin can cause PD like symptoms in human [11] through its metabolite MPP^+^ (N-methyl-4-phenylpyridinium) [12]. MPTP is a lipophilic molecule that can penetrate the blood-brain barrier. After being metabolized into MPP^+^ in astrocytes by monoamine oxidase B (MAO B), MPP^+^ enters the adjacent dopaminergic neurons in substantia nigra (SN), and mainly inhibits the activity of complex I of mitochondrial respiratory chain [6, 7, 13, 14]. MPTP-induced animal models, have been widely used to study PD pathogenesis and test treatment strategies for PD [12].

The yeast NDI1 (internal NADH-quinone oxidoreductase, NADH dehydrogenase), a single-subunit protein encoded by *NDI1* gene, can homologously replace the mammalian 45-subunit complex I [15, 16]. In this study, to examine the compensatory effect of yeast NDI1 protein on MPTP/MPP^+^-induced human complex I functional defects and cellular damage, a recombinant *NDI1* lentivirus was prepared, and MPP^+^ was used to establish a PD cell culture model. In the MPTP-induced mouse PD model, the therapeutic effect of *NDI1* gene therapy using recombinant adeno-associated virus rAAV-*NDI1* was evaluated in terms of mitochondrial function, histopathologic features and neurological behavior, as well as the therapy safety. This study suggests that yeast *NDI1* gene therapy may be used to treat sporadic PD and other diseases with complex I deficiency caused by MPTP like substances.

## Materials and methods

### Cell Lines and Cell Culture

Human neuronal cell line SH-SY5Y cells and viral packaging cell line 293T-17 cells (American Type Culture Collection) were cultured in Dulbecco’s modified Eagle medium (Gibco) supplemented with 10% fetal bovine serum (Gibco) at 37 ℃ under 5% CO_2_.

### Packaging of Recombinant Lentivirus

A recombinant lentiviral vector (pLVX-CMV-HA-*NDI1*-IRES-ZsGreen1) was constructed, with HA-tag inserted in the N-terminus of *NDI1* gene behind the mitochondrial targeting sequence. To produce the recombinant *NDI1* lentivirus, the recombinant lentiviral plasmids were co-transfected with packaging plasmids (pSPAX2 and pMD2G) into 293T-17 cells using the PEI (polyethylenimine) transfection protocol. The recombinant *NDI1* lentivirus supernatant was concentrated and purified using a method previously established by our research group before being used to transduce cells [17].

### MPP^+^ Treatment and Recombinant Lentivirus Transduction of SH-SY5Y Cells

The experimental groups include H_2_O + vector (control group), MPP^+^+vector (model group), and MPP^+^+NDI1 (therapy group). SH-SY5Y cells at 60–70% of confluence were transduced by the recombinant *NDI1* lentivirus or the lentivirus vector at multiplicity of infection (MOI) of 5 in condition of 8 µg/ml polybrene. Four days after viral transduction, SH-SY5Y cells were treated with 1 mM MPP^+^ (Sigma, D048) or H_2_O for 48 h before harvested for various assays. All of the experiments were repeated for three times.

### Cell Viability Assay

Cell viability was measured by staining dead cells with trypan blue. The culture supernatant, washing buffer, and cell suspension were all collected and centrifuged at 1,000 rpm for 5 min. The cell suspension was mixed with 0.4% trypan blue (ThermoFisher, 15,250,061) at 9:1. The blue dead cells and live cells were immediately counted.

### Immunofluorescence Staining

Cells were seeded on slides in 24-well plates and incubated with 100 nM Mito-Tracker Red CMXRos (ThermoFisher Scientific) at 37 °C for 30 min. The cells were then fixed with 4% paraformaldehyde for 30 min, permeabilized with 0.1% TritonX-100 for 10 min, and then blocked for 1 h. Subsequently, the cells were incubated with 1:100 HA antibody (cat#3724, Cell Signaling Technology) at 4 °C for 16–18 h, followed by incubating with Alexa fluo 647-labeled goat anti-mouse IgG antibody (1:500; Beyotime), and finally observed under a laser scanning confocal microscope (Nikon A1).

### Measurement of Mitochondrial Complex I-dependent Oxygen Consumption

Cells (5 × 10^6^) suspended in 2 ml detection solution (20 mM Hepes, 250 mM sucrose, 2 mM KH_2_PO_4_, 10 mM MgCl_2_, 1 mM ADP, pH = 7.1) were added to a chamber of Oxygraph-2k cell respiration instrument. After the cells permeabilized with 2% digitonin, 25µL of complex I substrate (1 M glutamic acid and 1 M malic acid mixed at 4:1) was first added to record the oxygen consumption of complex I. Then rotenone was added at a final concentration of 100 µM to inhibit mammalian endogenous complex I, but not to inhibit NDI1, and the oxygen consumption after inhibition was recorded. Subsequently, cells were treated with general complex I inhibitor flavone (6µL of 0.225 M), and the oxygen consumption after inhibiting the exogenous yeast complex I was recorded. Data analysis was performed with DatLab software.

### Measurement of ATP Content

ATP content was measured using the luciferin/luciferase ATP assay kit (ThermoFisher). Cells (10^6^) were lysed in ATP extraction buffer (100 mM Tris, 4 mM EDTA). Subsequently, the supernatant was mixed with the reaction solution, and the chemiluminescent detection was performed with multi-scan spectroscopy (ThermoFisher). The relative ATP level was calculated according to the standard curve. Besides the basal ATP production (Base) of untreated cells, as a parallel experiment, the cells were treated with 15 µg/mL oligomycin at 37 °C for 1 h, for determining the ATP production after treated with oligomycin. The oligomycin-sensitive ATP production and its ratio to basal ATP production were calculated.

### Determination of Mitochondrial ROS

Cells (5 × 10^5^) were incubated with 500 µL of 5 µM MitoSOX™ Red (mitochondrial superoxide indicator, ThermoFisher Scientific, USA) at 37 °C for 25 min, washed, and suspended for detection by flow cytometry (BD Biosciences). The median fluorescence intensity (MFI) was obtained from data of 5,000 cells for each sample.

### Animals for Experiment

All animal experiments were approved by the Animal Care and Use Committee of Wenzhou Medical University (approval number wydw2020-0804). All animal experiments complied with the ARRIVE guidelines and were carried out in accordance with the National Research Council’s Guide for the Care and Use of Laboratory Animals. C57BL/6 male mice (10–12 weeks old, weighing 20–25 g) were purchased from Shanghai Shrek Experimental Animal Co., Ltd.

### Establishment of MPTP-induced PD Mouse Model and Injection of rAAV5-*NDI1*

At least 24 mice for experiments were randomly divided into 4 groups: probenecid + vector (control group), probenecid + NDI1 (NDI1 safety group), MPTP + vector (model group) and MPTP + NDI1 (therapy group). Mice were injected intraperitoneally twice a week with 20 mg/kg MPTP or the probenecid as control for 5 weeks. Subsequently, the AAV5 vector alone or AAV5 expressing NDI1 (rAAV5-*NDI1*) virus (Wuhan Shumi Brain Science and Technology Co., Ltd. with a titer of 2.1 × 10^12^ vg/mL) was injected into SNpc of bilateral brain. Thirty-six to 41 days later, neurobehavioral experiments were conducted. On day 42, mice were euthanized to dissect brain samples for subsequent assays of pathology and mitochondrial function.

For stereotactic injection, mice were sedated with inhalation of 1.5% isoflurane, then fixed on a brain stereotaxic instrument (Stoelting). The rAAV5-*NDI1* (or empty vector) was injected using a Hamilton microsyringe attached with a glass tip. In the left and right cerebral hemispheres, 3 µL virus with a concentration of 2 × 10^12^ genome copies/mL, was separately injected at a rate of 0.6 µL/min into the following SN coordinates (bregma as the origin): AP (anteroposterior): −3.3 mm, ML (mediolateral): ±1.5 mm, and DV (dorsoventral): −3.9 mm. The injected amount of rAAV was according to previously published studies [18, 19]. Pain relievers and antibiotics were applied to the surface of the wound in each mouse.

### Pole Test

The pole test is used to evaluate rodent motor agility. Each mouse was placed head-upward at the bottom of a pole with a radius of 4 mm and a length of 50 cm. Then the time (T-turn) required for a mouse to complete a U-turn, and the total time (T-LA) required for a mouse to climb up, turn around and climb back down to ground, were recorded. The mice were trained continuously for 3 days, 3 times per day, and the testing experiment was performed on the 4th day, with 3–4 mice for each group.

### Rotarod Test

The rotarod test was used to evaluate the motor coordination ability of mice. The mice were continuously trained for 3 days, 10 min per day, with a rotational speed at 10 rpm. On the 4th day, the testing experiment was implemented with a rotational speed at 40 rpm, and the latency time before falling from a rotarod was recorded, with 4–6 mice for each group.

### Open Field Test

The open field test was used to evaluate the exploratory behavior of mice. The operation box used in this study had a 40 cm × 40 cm open field at its base, over which there was a camera connected to EthoVision XT recording and analyzing system to track the movement of a mouse in the open field within 15 min. Within the open field, an area of 20 cm × 20 cm was set as the central area, the total movement distance and the ratio of central movement distance to total movement distance were counted and analyzed (with 3–5 mice for each group).

### Western Blot

For cultured cell samples, the culture supernatant, washing buffer and cell suspension were all harvested. The pelleted cells were lysed in RIPA buffer and centrifuged. The supernatants were separated by SDS-PAGE and transferred to PVDF membrane. The membrane was first incubated with anti-human HA antibody (1:100; cat# 3724, Cell Signaling Technology), or cleaved caspase-9 antibody (1:1000; cat# 9508, Cell Signaling Technology), or cleaved caspase-3 antibody (1:1000; cat# 9661, Cell Signaling Technology), or β-actin antibody (1:2000; cat# TA811000, OriGene) overnight at 4 °C, and subsequently with HRP-conjugated appropriate secondary antibody. The protein bands were developed with ECL reagent, detected by the ChemiDoc MP imaging system (Bio-Rad), and were analyzed using the Image Lab 5.0 software (Bio-Rad). In animal experiments, after mice were euthanized, SN and striatum tissues from both sides of the brain were dissected and lysed in RIPA lysis buffer. The remaining steps were the same as that for cultured cell samples, except for the primary antibody being mouse anti-HA antibody (Beyotime) or anti-GAPDH antibody (Proteintech).

### H&E (Hematoxylin-Eosin) Staining and Immunohistochemistry

SN and striatum tissues from paraformaldehyde-perfused mouse brain tissues were fixed in 4% formaldehyde at 4 °C overnight. H&E staining of slices was performed with H&E staining kit (Beijing Solarbio). The immunohistochemical methods have been previously described by our research group [20]. The primary antibodies used in this study included anti-mouse tyrosine hydroxylase (TH) antibody (1:1000, Immun Star), anti-mouse GFAP antibody (1:1000, Abcam), anti-mouse Iba-1 antibody (1:1000, Abcam) or anti-mouse NeuN antibody (1:1000, Sigma). Three fields were taken randomly from one slice of four different slices for each group, and the average value was calculated.

### Determination of Overall Mitochondrial Oxygen Consumption

For cultured cell samples, 5 × 10^6^ cells were added into a chamber of Oxygraph-2k cell respiration apparatus, containing 2 ml buffer (25 mM Tris-HCl, 137 mM NaCl, 10 mM KCl, 0.7 mM Na_2_HPO_4_, pH = 7.4). The basal oxygen consumption of untreated cells was firstly recorded, ATP synthase inhibitor oligomycin was subsequently added at a final concentration of 2.5 µg/mL to measure the inhibited oxygen consumption. Finally, the respiratory chain uncoupler FCCP [carbonyl cyanide 4-(trifluoromethoxy) phenylhydrazone] was added at a final concentration of 0.1 µM to measure the maximal oxygen consumption. For mouse tissue samples, fresh SN tissues (from 4 mice for each group) from both sides of brain were dissected as the above. The methods of oxygen consumption measurement were previously described by our research group [20]. Data analysis was performed with DatLab software.

### Measurement of Mitochondrial Complex I Enzyme Activity

Fresh brain SN tissues from both sides of brain were taken. The methods of mitochondrial complex I enzyme activity measurement were previously described by our research group [20]. Briefly, tissue samples were homogenized and centrifuged to obtain highly purified mitochondria. The isolated mitochondria were resuspended and repeatedly frozen and thawed. The rate of NADH oxidation in a reaction mixture was detected by a spectrophotometer, reflecting complex I enzyme activity. The complex I enzyme activity was normalized by the citrate synthase activity. The enzyme activity measurement of each mitochondrial specimen was repeated for 3 times. There were 3 mice for each group.

### Statistical Analysis

The quantity data were expressed as mean ± standard deviation, and the comparison of mean values among groups was performed using one-way ANOVA in SPSS 22.0 software. The homogeneity of variances was first estimated. If the variances were homogeneous, *P* value was calculated by Tukey’s post hoc test. If the variances were not homogeneous, *P* value was calculated by Tamhane’s T2. *P* < 0.05 was considered statistically significant.

## Results

### *NDI1* Gene was Efficiently Expressed and Located in Mitochondria after Transduced into SH-SY5Y Cells

The recombinant *NDI1* lentivirus was used to transduce SH-SY5Y cells. The percentage of GFP (NDI1)-positive cells was 94.6% (Supplementary Fig. 1A). On the 5th and 8th day after transduction, the expression of HA-tagged NDI1 in transduced SH-SY5Y cells was at a high level detected by Western blot (Supplementary Fig. 1B), which was sufficient to have a therapeutic effect during this period. Furthermore, the co-localization of HA (NDI1) with MitoTracker was observed (Supplementary Fig. 1C). These results demonstrated that NDI1 protein was properly expressed and located in the mitochondria of SH-SY5Y cells.

### NDI1 can Resist the Morphological Changes and the Decrease of Cell Survival in MPP^+^-induced PD Cell Culture Model

**Fig. 1:**
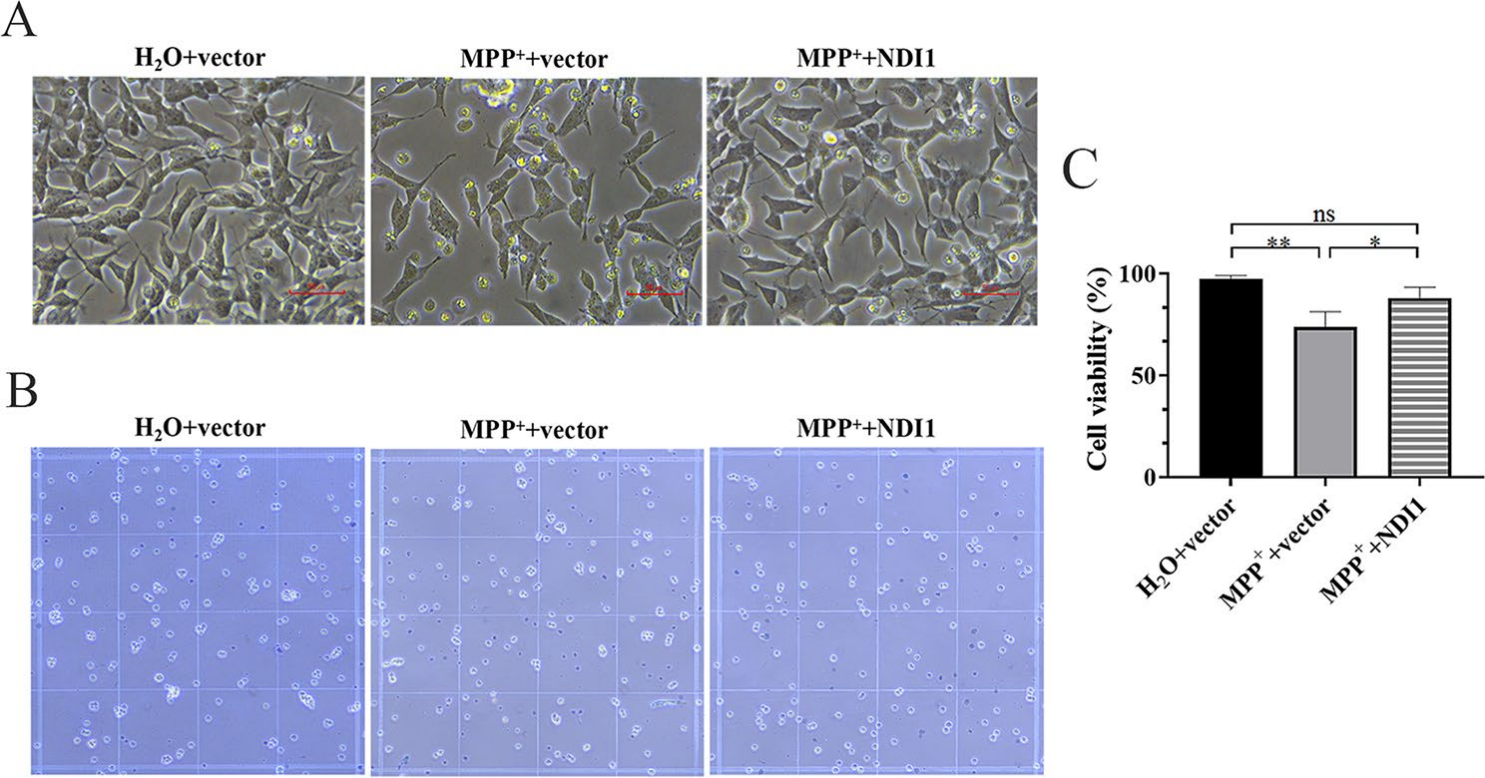
NDI1 can resist the morphological changes and the decrease of cell survival in MPP^+^-induced PD cell culture model. **A**: Cell morphology was observed with microscope (scale bar: 50 μm). **B**: Cell viability was determined with trypan blue staining. **C**: Statistical analysis of cell viability. ns: no significant difference, *: *P* < 0.05, **: *P* < 0.01

Microscopy examination revealed that MPP^+^ treatment induced an abnormal change in cell morphology such as reduced cell protrusions and swollen cell shape in MPP^+^+vector group, whereas in MPP^+^+NDI1 group, the cell morphology was essentially normal compared with H_2_O + vector group (Fig. 1A). In addition, results from trypan blue staining (Fig. 1B) showed that the cell viability of MPP^+^+vector group was lower than that of either H_2_O + vector group (*P* < 0.01) or MPP^+^+NDI1 group (*P* < 0.05) (Fig. 1C). There was no significant difference between MPP^+^+NDI1 group and H_2_O + vector group. These results indicated that NDI1 can resist morphological changes and cell death caused by MPP^+^ in PD cell culture model.

### NDI1 can Partially Restore the Oxidative Phosphorylation Function of Mitochondria in MPP^+^-induced PD Cell Culture Model

**Fig. 2:**
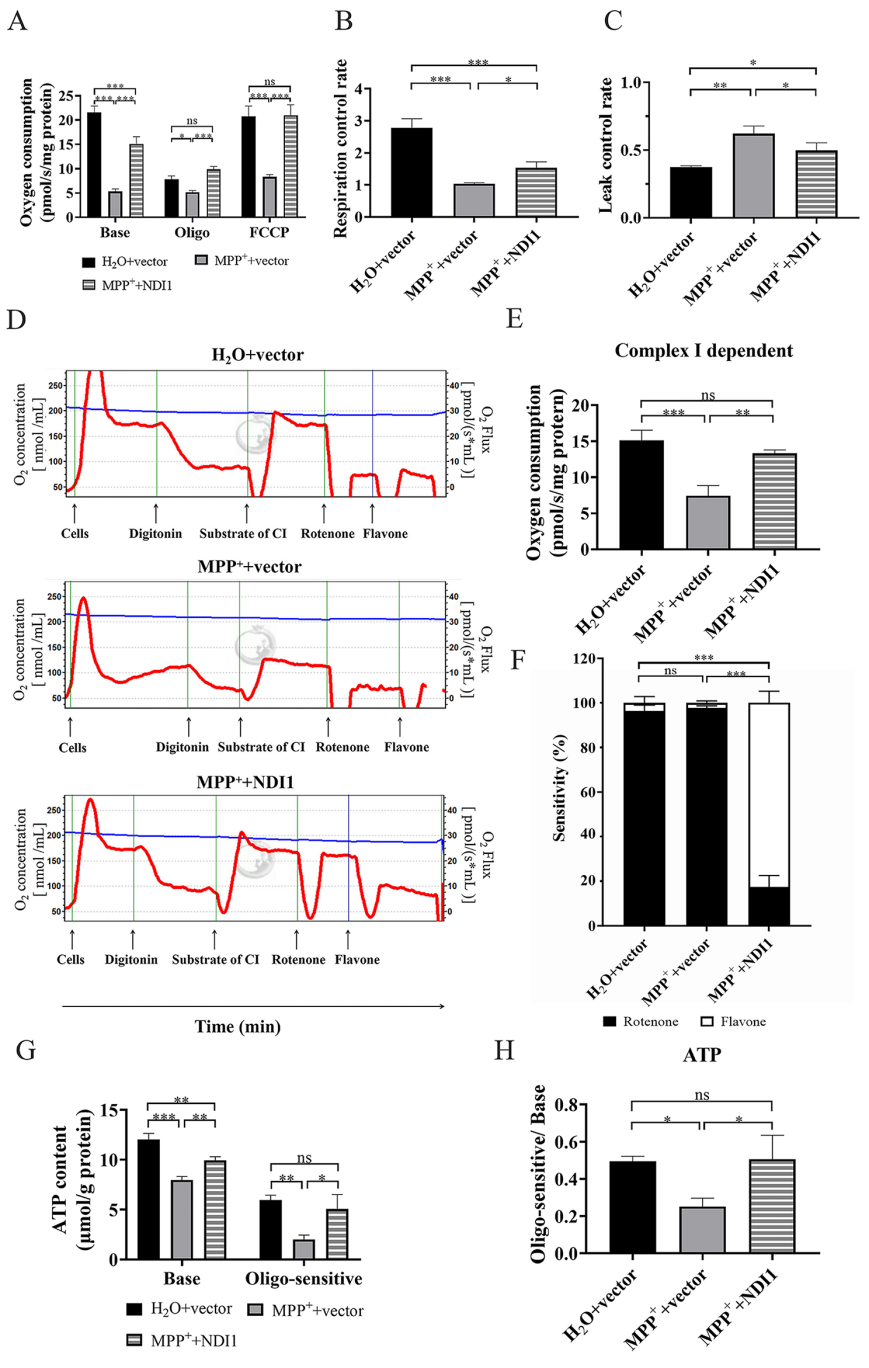
NDI1 can partially restore the oxidative phosphorylation function of mitochondria in MPP^+^-induced PD cell culture model. **A**: The basal (Base), oligomycin-treated (Oligo), and FCCP-treated (FCCP) oxygen consumption. **B**: RCR (respiratory control rate) calculated by Base/Oligo. **C**: LCR (leakage control rate) calculated by Oligo/FCCP. **D**: Complex I-dependent oxygen consumption detected by cellular respiration apparatus. **E**: Statistical analysis of complex I-dependent oxygen consumption. **F**: The proportion of rotenone or flavone sensitive in complex I-dependent oxygen consumption. G: The basal ATP production (Base) and oligomycin-sensitive ATP production (Oligo-sensitive). H: The ratio of Oligo-sensitive ATP production. ns: not significant, *: *P* < 0.05, **: *P* < 0.01, ***: *P* < 0.001

To verify the alternative compensation effect of NDI1 on MPP^+^-induced defective mitochondrial oxidative phosphorylation, the overall oxygen consumption level, complex I-dependent oxygen consumption level, and ATP level were measured. For the overall mitochondrial oxygen consumption, the basal oxygen consumption in MPP^+^+vector group was decreased significantly in comparison with H_2_O + vector group (*P* < 0.001), whereas it was significantly higher in MPP^+^+NDI1 group than in MPP^+^+vector group (*P* < 0.001)(Fig. 2A, Base). After ATP synthase (complex V) was inhibited by oligomycin, the oxygen consumption was lower significantly in MPP^+^+vector group than in H_2_O + vector group (*P* < 0.05) or MPP^+^+NDI1 group (*P* < 0.001), and MPP^+^+NDI1 group had a completely recovered level in comparison with H_2_O + vector group (Fig. 2A, Oligo). The result of maximum oxygen consumption (Fig. 2A, FCCP), detected after the respiratory chain uncoupler FCCP was added, was similar as Oligo (Fig. 2A, Oligo). The results indicated NDI1 could restore MPP^+^-impaired oxygen consumption levels to a certain extent. Furthermore, the respiratory control rate (RCR) and leakage control rate (LCR) were used to respectively evaluate the oxidative phosphorylation coupling efficiency and proton leakage of mitochondria. RCR in MPP^+^+vector group was significantly lower than that in H_2_O + vector group (*P* < 0.001), and it was increased in MPP^+^+NDI1 group than in MPP^+^+vector group (*P* < 0.05) (Fig. 2B). LCR was higher in MPP^+^+vector group than H_2_O + vector group (*P* < 0.01) and MPP^+^+NDI1 group (*P* < 0.05) (Fig. 2C).

The complex I-dependent oxygen consumption was examined after the cells were permeabilized with digitonin and the complex I substrate (malic acid + glutamic acid) was added (Fig. 2D). Complex I-dependent oxygen consumption was significantly lower in MPP^+^+vector group than in H_2_O + vector group (*P* < 0.001) and MPP^+^+NDI1 group (*P* < 0.01), and there was no significant difference between H_2_O + vector group and MPP^+^+NDI1 group (Fig. 2E). These results indicated that NDI1 could compensate for complex I-dependent oxygen consumption damage caused by MPP^+^. Moreover, the contribution ratios of endogenous mammalian complex I or exogenous yeast complex I (NDI1) to total complex I-dependent oxygen consumption were investigated by determining the sensitivity to specific complex I inhibitors. Endogenous mammalian complex I-dependent oxygen consumption is sensitive to rotenone, whereas exogenous yeast NDI1-dependent oxygen consumption is insensitive to rotenone but sensitive to flavone. The specific mammalian complex I inhibitor rotenone was first added, and then the general complex I inhibitor flavone. The most part of (nearly all) complex I-dependent oxygen consumption was sensitive to rotenone in H_2_O + vector group, as well as in MPP^+^+vector group. In MPP^+^+NDI1 group, only small part of complex I-dependent oxygen consumption was sensitive to rotenone while large part was sensitive to flavone (Fig. 2F), indicating that NDI1 was the main player and substituted for a large proportion of impaired endogenous complex I function in transduced cells.

Analysis of the cellular ATP levels showed that the baseline ATP level of MPP^+^+vector group was significantly lower than that of H_2_O + vector group (*P* < 0.001), it was increased in MPP^+^+NDI1 group in comparison with MPP^+^+vector group (*P* < 0.01) (Fig. 2G, Base). In addition, the part of ATP production sensitive to oligomycin, which represents the ATP produced by mitochondria, was detected (Fig. 2G, Oligo-sensitive). In MPP^+^+vector group this part of ATP production was lower than that of H_2_O + vector group (*P* < 0.01), while in MPP^+^+NDI1 group it was increased (*P* < 0.05) and recovered to the level of H_2_O + vector group. The result of the proportion of oligomycin-sensitive ATP production, calculated by Oligo-sensitive/Base (Fig. 2H), was consistent with that of Oligo-sensitive (Fig. 2G). The above results indicated that NDI1 could almost completely restore impaired mitochondrial oxidative phosphorylation caused by MPP^+^.

### NDI1 can Reduce Mitochondrial ROS Content and Resist Mitochondrion-mediated Apoptosis in MPP^+^-induced PD Cell Culture Model

**Fig. 3:**
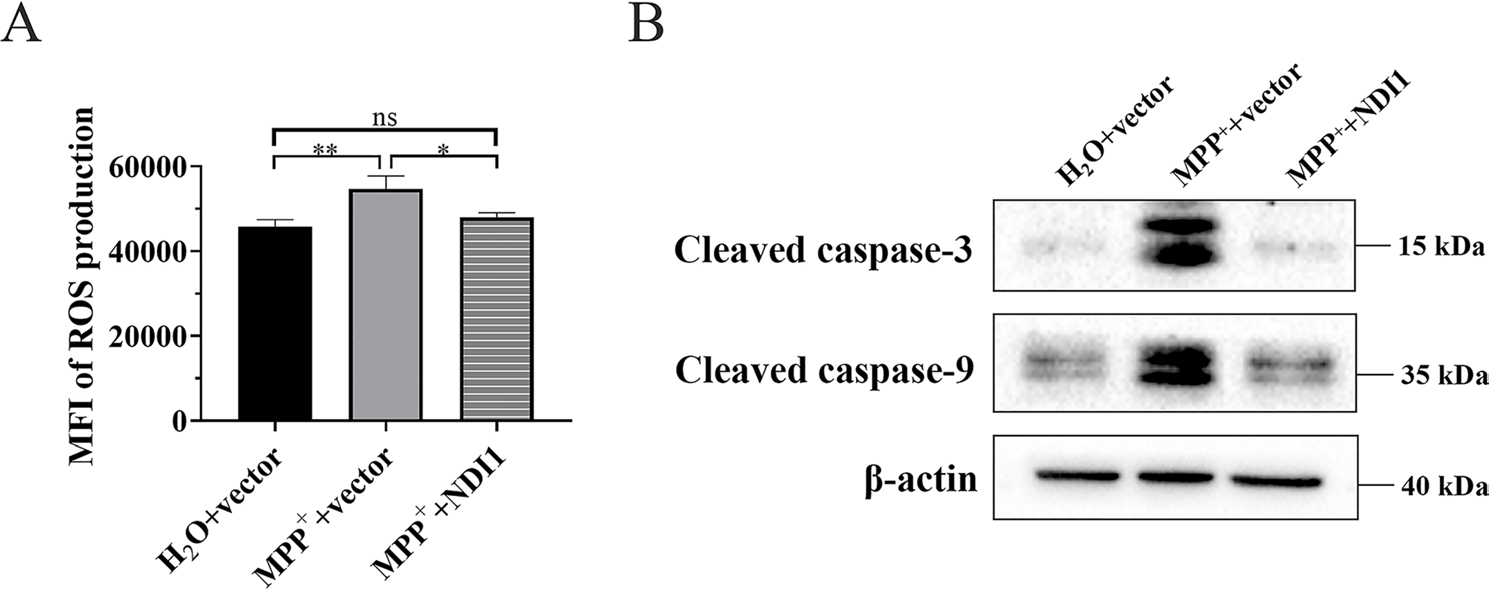
NDI1 can reduce mitochondrial ROS content and resist mitochondrion-mediated apoptosis in MPP^+^-induced PD cell culture model. **A**: The mitochondrial ROS production detected by flow cytometry using MitoSOX™ staining. **B**: The level of mitochondrion-mediated apoptosis was examined by Western blot. ns: no significant difference, *: *P* < 0.05, **: *P* < 0.01

The mitochondrial ROS content (Fig. 3A) of MPP^+^+vector group was higher than that of H_2_O + vector group (*P* < 0.01), while in MPP^+^+NDI1 group it was decreased to that of H_2_O + vector group. These results indicated that MPP^+^ can increase the mitochondrial ROS content, while NDI1 can reduce the level of oxidative stress.

Neuronal cell death in SN, the major pathological change in PD, may primarily happen through aberrantly activated mitochondrion-mediated (intrinsic) apoptosis. Compared with H_2_O + vector group, cleaved caspase-9 and cleaved caspase-3 levels in MPP^+^+vector group were obviously increased, but were not increased in the MPP^+^+NDI1 group (Fig. 3B). These results indicated that MPP^+^ can induce excessive apoptosis through mitochondrial pathway in PD cell culture model, which can be rescued by the transduction of *NDI1*.

### NDI1 was Efficiently Expressed in Bilateral SN of MPTP-Induced PD Mouse Model

**Fig. 4:**
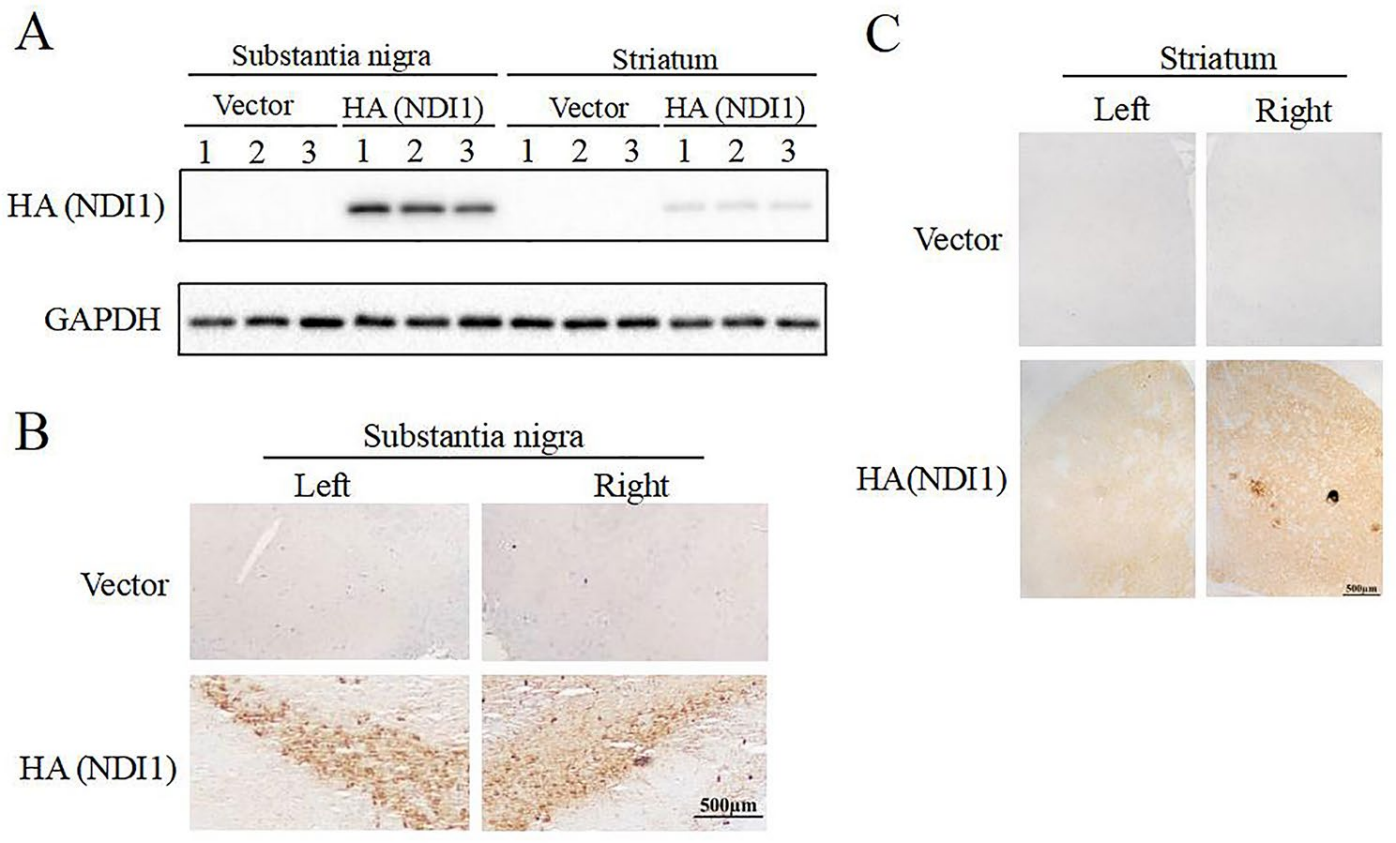
NDI1 was efficiently expressed in bilateral SN of MPTP-induced PD mouse model. **A**: The HA (NDI1) expression in SN and striatum was examined by Western blot. **B**: The HA (NDI1) expression in SNpc was shown by immunohistochemical staining (scale bar: 500 μm). **C**: The HA (NDI1) expression in striatum was shown by immunohistochemical staining (bar: 500 μm)

The expression of rAAV-*NDI1* in SN and striatum was examined by western blot. In MPTP + NDI1 group, the HA-tagged NDI1 protein was detected, indicating a strong expression in SN but a weak expression in striatum (Fig. 4A). Immunohistochemical staining further revealed that NDI1 protein was expressed in SNpc and striatum from both left and right side brain in MPTP + NDI1 group (Fig. 4B and C).

### NDI1 can Improve the Neurobehavioral Function in MPTP-Induced PD Mouse Model

**Fig. 5:**
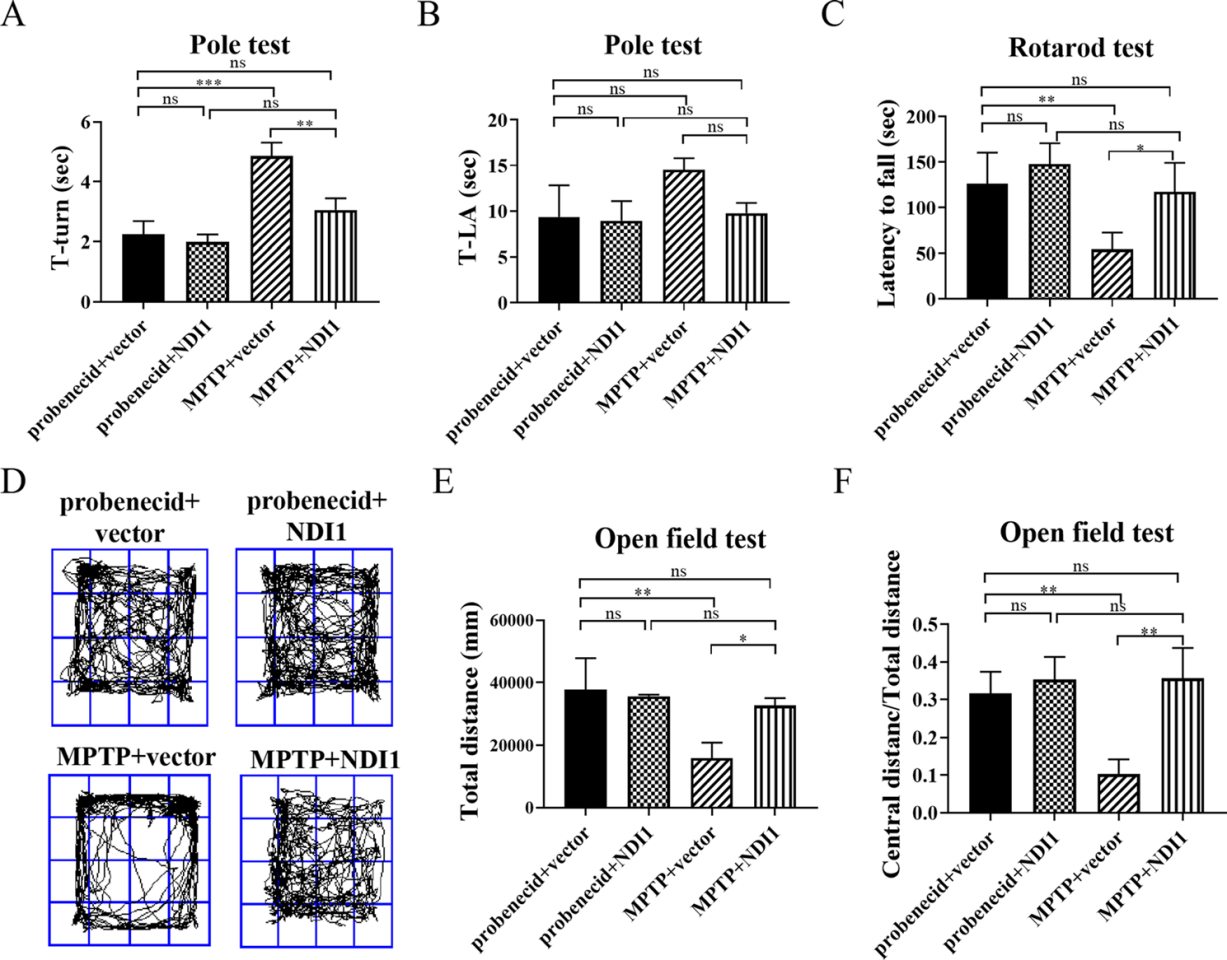
NDI1 can improve the neurobehavioral function in MPTP-induced PD mouse model. **A**: The time (T-Turn) required for mouse to turn around completely in pole test. **B**: The total time (T-LA) required for mouse to climb up, turn around and climb down to the ground in pole test. **C**: The latency time before fall (Latency to fall) in rotarod test. **D**: The movement trajectory in open field test. **E**: Quantitative statistical analysis of total distance in open field test. **F**: Statistical analysis of the ratio of central movement distance to total movement distance in open field test. ns: no significant difference, *: *P* < 0.05, **: *P* < 0.01, ***: *P* < 0.001

In order to explore the rehabilitation efficacy of NDI1, neurobehavioral tests were conducted to evaluate the motor agility, motor coordination and exploratory behaviors of mice. In pole test, T-turn (the time required to complete U-turn) was significantly prolonged in MPTP + vector group (Fig. 5A, *P* < 0.001 or *P* < 0.01) in comparison with probenecid + vector or MPTP + NDI1 group. In the rotarod test (Fig. 5C), the latency time before fall was shorter in MPTP + vector group than in probenecid + vector (*P* < 0.01) or in MPTP + NDI1 group (*P* < 0.05). The results showed that the neurotoxin MPTP impaired the motor function of mice, and NDI1 could improve the motor function in affected mice. In the open field test, both total movement distance and the ratio of central movement distance to total movement distance, were decreased in MPTP + vector group in comparison with probenecid + vector (Fig. 5D, E, F, *P* < 0.01) or MPTP + NDI1 group (Fig. 5D, E, F, *P* < 0.05 or *P* < 0.01). These results indicated that MPTP treatment caused impairment of motor ability and exploring behavior, which could be improved by NDI1 in mice.

### NDI1 can Maintain Normal Morphology and Survival of Neurons in SN of MPTP-induced PD Mouse Model

**Fig. 6:**
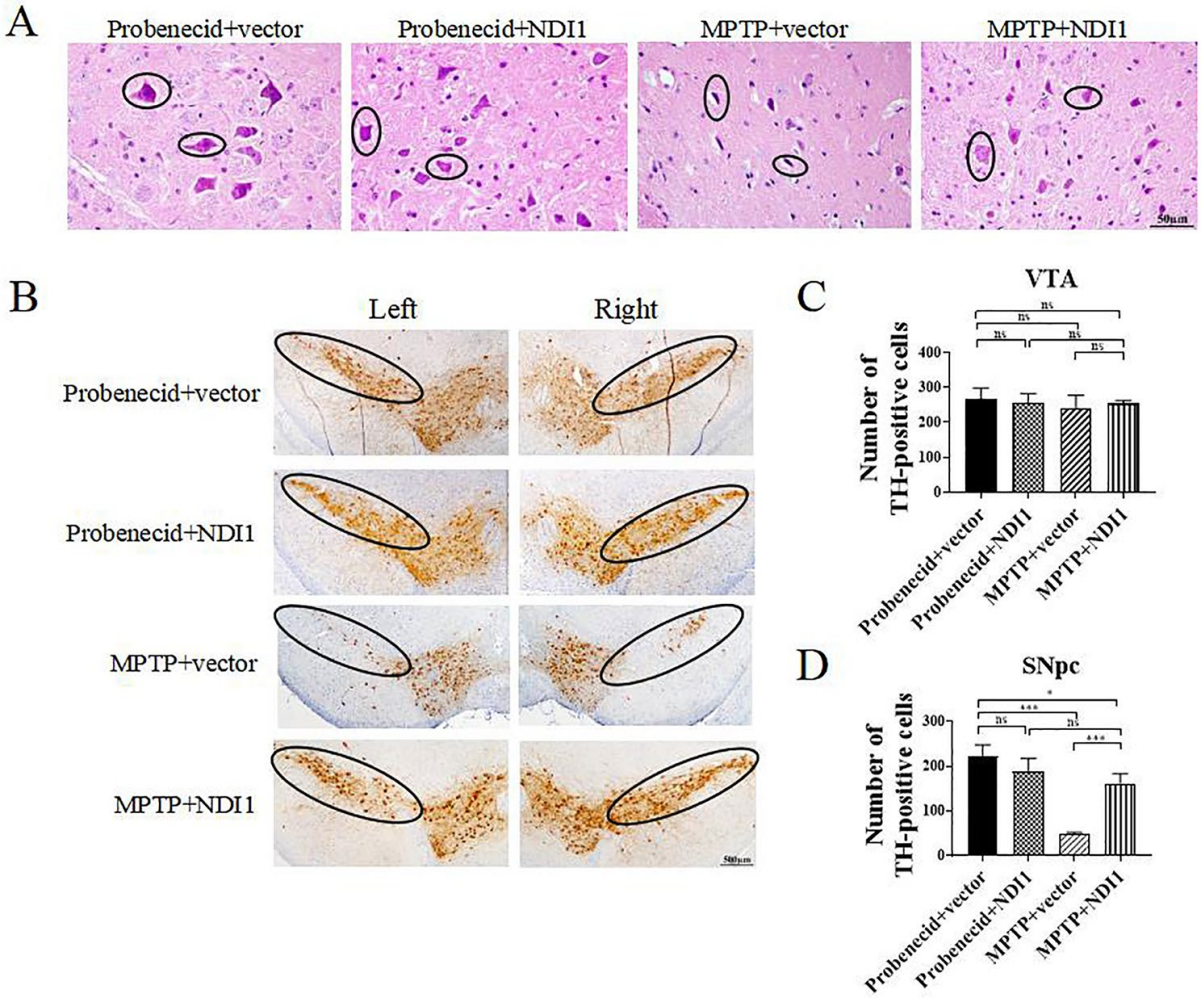
NDI1 can maintain normal morphology and survival of neurons in SN of MPTP-induced PD mouse model. **A**: The morphology of neurons in SN (scale bar: 50 μm). **B**: Tyrosine hydroxylase (TH)-positive surviving neurons in SNpc (indicated by black circle) and ventral tegmental area (VTA) (scale bar: 500 μm). **C**, **D**: Statistical analysis of surviving number of neurons in VTA and SNpc. ns: no significant difference, *: *P* < 0.05, ***: *P* < 0.001

The mainly pathological changes of PD are characterized by degeneration and death of neurons in SNpc. Firstly, morphological examination of neurons in SNpc revealed that MPTP induced degenerated morphology of dopaminergic neurons, which could be largely normalized by NDI1 (Fig. 6A). Subsequently, the survival of neurons in SNpc was evaluated by immunohistochemical staining, using tyrosine hydroxylase (TH), the rate-limiting enzyme controlling the synthesis of dopamine, as an indicator of viable dopaminergic neurons. The distribution region of dopaminergic neuron in SNpc appeared as an inverted eyebrow on tissue sections (Fig. 6B). MPTP hardly damaged dopaminergic neurons in VTA (Fig. 6C). The number of surviving dopaminergic neurons in SNpc was significantly decreased in MPTP + vector group in comparison with probenecid + vector group (*P* < 0.001), or MPTP + NDI1 group (*P* < 0.001), indicating that NDI1 can repair MPTP-induced damage and maintain the survival of dopaminergic neurons (Fig. 6D).

### NDI1 can Restore Dopamine Content in Striatum of MPTP-induced PD Mouse Model

**Fig. 7:**
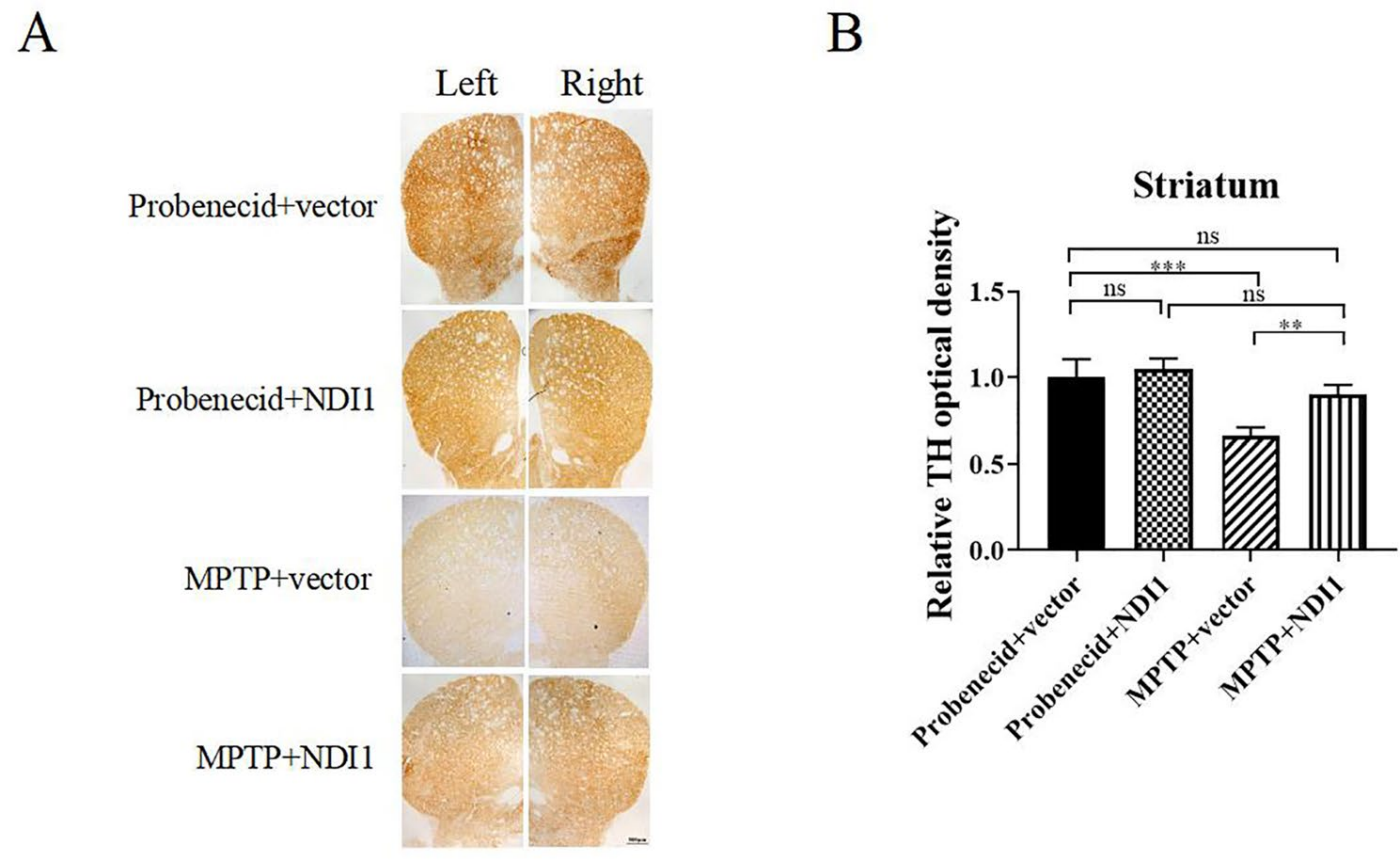
NDI1 can restore TH content (indicating dopamine content) in striatum of MPTP-induced PD mouse model. **A**: Tyrosine hydroxylase (TH) content in striatum examined by immunohistochemical staining with TH antibody (brown) (scale bar: 500 μm). **B**: Statistical histogram from A displaying TH-positive optical density value in striatum. ns: no significant difference, **: *P* < 0.01, ***: *P* < 0.001

TH immunohistochemical staining in striatum can reflect dopamine content in striatum. The TH staining in left and right striatum suggested that the dopamine content in striatum was reduced by MPTP (Fig. 7A and B, *P* < 0.001). In MPTP + NDI1 group, the TH staining was higher than that in MPTP + vector group (Fig. 7A and B, *P* < 0.01), indicating that the transduction of *NDI1* in SNpc can increase the dopamine content in striatum.

### NDI1 can Inhibit Inflammatory Response and Maintain Number of Neurons in SN of MPTP-induced PD Mouse Model

**Fig. 8:**
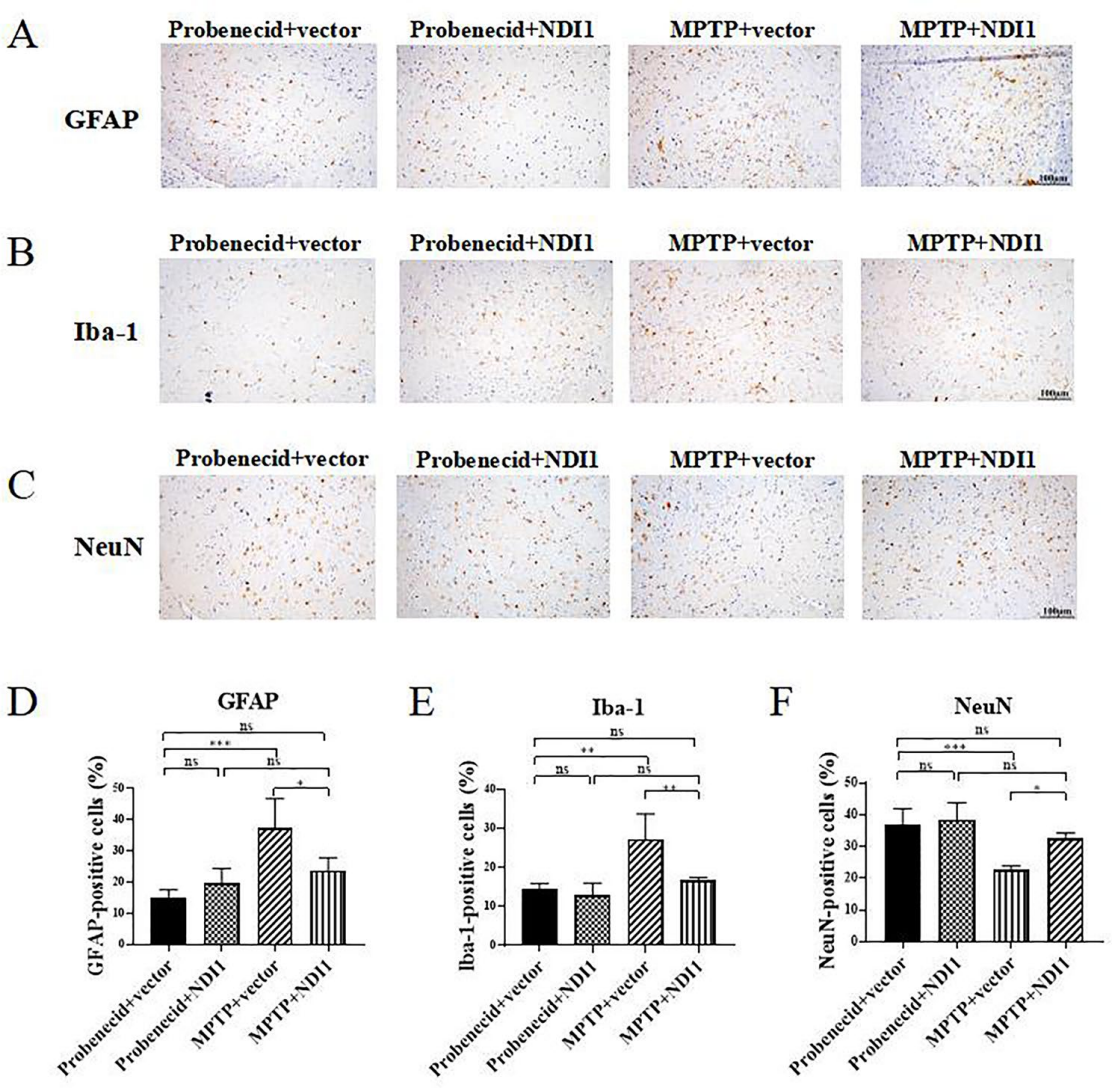
NDI1 can inhibit inflammatory response (decrease the number of astrocytes and microglia) and maintain number of neurons in SN of MPTP-induced PD mouse model. **A**, **B**, **C**: Astrocytes (GFAP-positive, cytoplasm), microglia (Iba-1-positive, cytoplasm), neurons (NeuN-positive, nucleus) were detected by immunohistochemical staining with GFAP antibody, Iba-1 antibody and NeuN antibody, respectively. (Scale bar: 100 μm). **D**, **E**, **F**: Statistical analysis of the proportion of astrocytes (GFAP-positive), microglia (Iba-1 positive), neurons (NeuN-positive). ns: no significant difference, *: *P* < 0.05, **: *P* < 0.01, ***: *P* < 0.001

To explore the inflammatory response in SN, the abundances of astrocytes (GFAP-positive) and microglia (Iba-1-positive) were examined (Fig. 8A and B). The proportions of both astrocytes (GFAP-positive) and microglia (Iba-1-positive) in SN were significantly increased in MPTP + vector group in comparison with probenecid + vector group (*P* < 0.001, *P* < 0.01) (Fig. 8D and E), indicating that MPTP generated inflammatory response by increasing astrocytes and microglia. In MPTP + NDI1 group, the proportions of these two types of inflammatory responsive cells were lower than that in MPTP + vector group (*P* < 0.05, *P* < 0.01) (Fig. 8D and E), indicating that *NDI1* transduction in SNpc can inhibit the occurrence of MPTP-induced inflammatory response.

The expression of NeuN, the most commonly used neuronal marker, in SN was examined by immunohistochemistry (Fig. 8C). The percentage of NeuN-positive cells in the SN was significantly lower in MPTP + vector group than in probenecid + vector group (Fig. 8F, *P* < 0.001), indicating that MPTP damaged neurons in SN. The percentage of NeuN-positive cells was higher in MPTP + NDI1 group than in MPTP + vector group (*P* < 0.05), indicating that *NDI1* transduction in SNpc can inhibit MPTP from damaging neurons.

### NDI1 can Compensate for the Impaired Oxidative Phosphorylation Function in SN of MPTP-induced PD Mouse Model

**Fig. 9:**
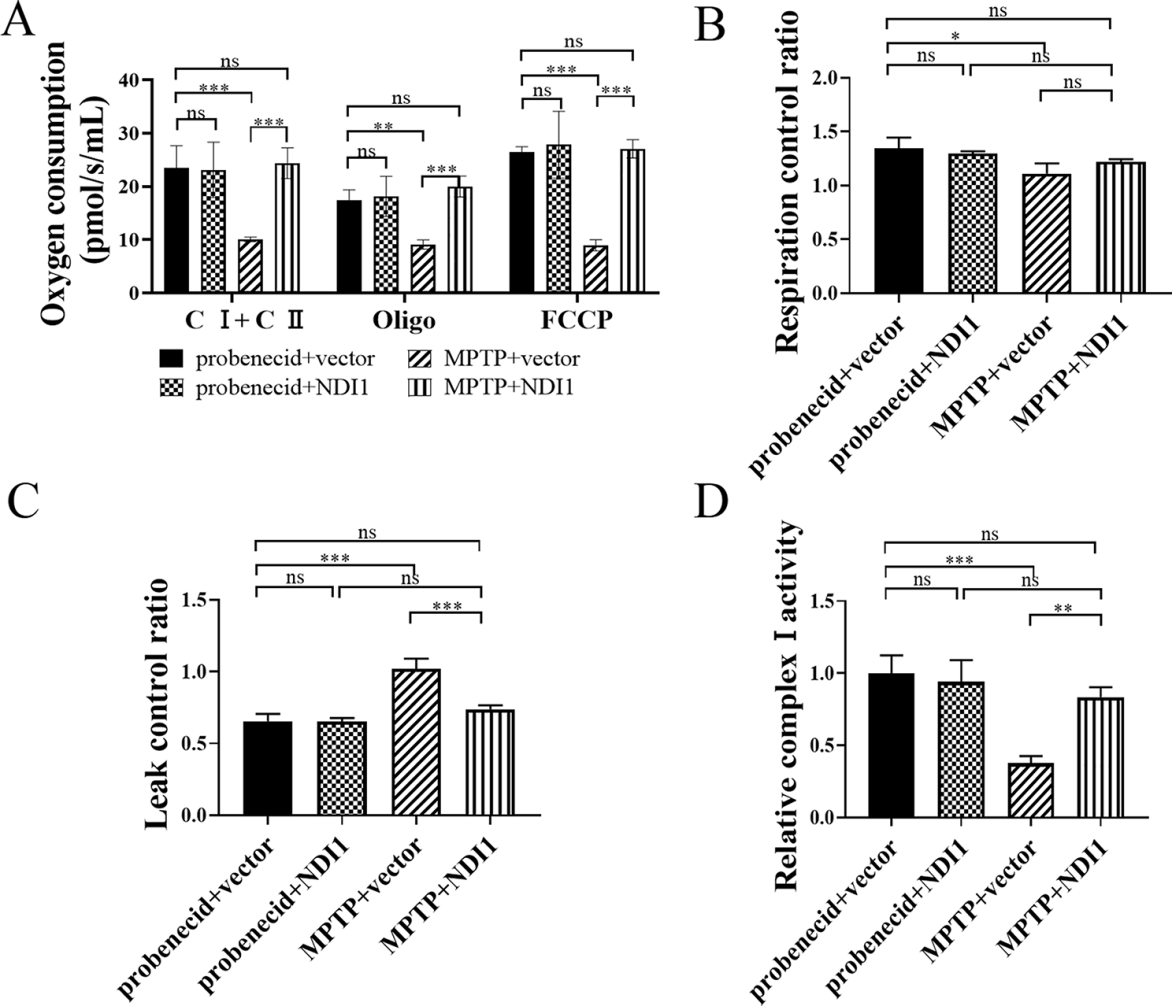
NDI1 can compensate for the impaired mitochondrial oxidative phosphorylation in SN of MPTP-induced PD mouse model. **A**: The mitochondrial total oxygen consumption of complex I and complex II (CI+CII), the oligomycin-treated and FCCP-treated oxygen consumption. **B**: RCR (respiratory control rate) was calculated by (CI+ CII)/Oligo. **C**: LCR (leakage control ratio) was calculated by Oligo/FCCP. **D**: The mitochondrial complex I enzyme activity in SN was detected by measuring NADH oxidation rate. ns: no significant difference, *: *P* < 0.05, **: *P* < 0.01, ***: *P* < 0.001

The mitochondrial total oxygen consumption level of complex I and complex II (C I + C II), the oxygen consumption levels after treated with oligomycin (Oligo) or FCCP (FCCP), were all reduced in MPTP + vector group in comparison with probenecid + vector group (Fig. 9A, *P* < 0.001, *P* < 0.01, *P* < 0.001), whereas these levels were significantly elevated in MPTP + NDI1 group in comparison with MPTP + vector group (*P* < 0.001). RCR was decreased in MPTP + vector group (*P* < 0.05), slightly but not significantly decreased in MPTP + NDI1 group, in comparison with probenecid + vector group (Fig. 9B). LCR was markedly higher in MPTP + vector group than in probenecid + vector group (Fig. 9C, *P* < 0.001), whereas in MPTP + NDI1 group it was maintained at normal level as probenecid + vector group. The results indicated that the defects in mitochondria-related respiration function in MPTP-induced PD mouse model can be restored by *NDI1* transduction.

To verify the role of mitochondrial complex I in SN of MPTP-induced PD mouse model, the activity of mitochondrial complex I was detected by NADH oxidation rate. The relative activity of mitochondrial complex I enzymes was significantly decreased in MPTP + vector group in comparison with probenecid + vector group (*P* < 0.001) or in MPTP + NDI1 group (*P* < 0.01) (Fig. 9D). The results indicated that the endogenous complex I activity in SN was impaired by MPTP treatment, and exogenous NDI1 can compensate for the activity of mitochondrial complex I.

## Discussion

Deficiencies in mitochondrial complex I contribute to the pathological features and clinical symptoms of PD, which still lacks effective cure currently. Human mitochondrial complex I, consisted of 45 subunits encoded by nuclear genome and mitochondrial genome, are susceptible to congenital or acquired environmental insults. In this study, we found that transduction of virus expressing yeast NDI1 protein has therapeutic efficacy against PD in MPP^+^/MPTP-induced PD cell and mouse models. MPTP causes PD like symptoms in human [11] through its metabolite MPP^+ (12)^, which can inhibit the activity of mitochondrial complex I in dopaminergic neurons. Although rotenone exposure has been associated to increased PD risk, there is no direct evidence that rotenone causes PD like disease in human. Although both MPTP and rotenone can inhibit the activity of mitochondrial complex I, there are differences in their delivery ways into neurons and their intracellular action sites [21]. Our previous study showed that the pathological features of PD, such as loss of dopaminergic neurons in SN, decreased dopamine level, and neuroinflammation response, were better reproduced in MPTP-induced PD mouse model than in rotenone-induced [20].

It is worth pointing out that the strategy of rAAV-*NDI1* delivery into SN of PD model mice in this study has been improved to provide solid evidences of achieving effective efficacy. One improvement was to imitate the chronic pathogenesis of PD patients by generating PD mouse model through chronic administration of MPTP into mice. In previous studies, MPTP was administrated acutely, with rAAV being injected 2–4 weeks before the MPTP administration [22–24]. More importantly, in this study, the rAAV was injected at the same day when MPTP was administrated, which made the peak expression of rAAV-directed *NDI1* (day 35–42) occur after the appearance of MPTP-induced PD symptoms (day 1–35). Although this method was hard to carry out, with a higher risk of mouse fatality since the brain stereotaxic injection was conducted in the mice that had been injured by MPTP toxicity, it is more closely conformed to the principal that gene therapy should conduct after the PD animal model has been established. This is the major difference of our study from three published reports that used yeast NDI1 to prevent the onset of PD mouse model induced by MPTP [25–27]. The second merit of this study is the design of bilateral SN injection of *rAAV-NDI1*. Bilateral SN treatment is a prerequisite for many neurobehavioral tests such as pole test, rotarod test, and open field test, used in PD mouse model study. More importantly, the clinical trials are all implemented by bilateral treatments [28]. Since bilateral brain stereotaxic injection requires higher operational skill to reduce the injury and inflammation, unilateral injections were reported in some studies [24–27].

The current molecular and cellular therapy strategies under development for PD include stem cell transplantation [29–31], antibody injection, and gene therapy approach including viral vector-mediated gene transduction, RNA interference, and gene editing, etc [28, 32–35]. Theoretically, gene therapy is an ideal and curative approach [5]. Up to now, two main types of target genes have been investigated for PD gene therapy. The first type consists of protective genes including neurotropic factor genes *GDNF* and *NRTN*, anti-apoptosis and anti-free radical genes, which are expected to protect dopaminergic neurons from damage factors [33, 36, 37]. Another type includes genes encoding enzymes involving in dopamine metabolism, such as three enzymes AADC, TH, and GCH, for promoting the efficient production of dopamine [28, 32, 33, 38–40]. Although many gene therapies for PD have shown promising results in preclinical models, only a handful have progressed to clinical trials. Nevertheless, for these completed or underway Phase I and Phase II clinical trials, including those for AAV2-*GAD* [41], AAV2-*GDNF* [34, 40, 42], AAV2-*AADC* [28, 38, 43] and AAV2-*NRTN* [44], the curative effects have not been very satisfactory [34, 45], with only a few advancing to Phase III clinical trials [46–48].

Mitochondrial dysfunction can lead to diseases in many tissues, especially those with high energy requirements such as nerve and muscle tissues. Since human mitochondrial complex I contains as many as 45 subunits, the accumulation of structural or functional abnormalities on different individual subunit of complex I in cells may impair the main function of mitochondria, ultimately leading to diseases. There have been three main strategies targeting the defective mitochondrial complex I. One is the mitochondrial transplantation, which has the drawback of low efficiency of transplantation [49]. The other one is to replace with cell-permeable proteins, but the preparation, purification and storage of proteins are far from the requirements of clinical treatment [50]. The third one is gene therapy targeting complex I, such as the preclinical research and clinical trial for Leber’s hereditary optic neuropathy targeting *ND4* gene [51, 52] and the gene therapy by AAV-*Ndufs4* in Leigh syndrome mouse model of systemic *Ndufs4*-knockout [53]. The above gene therapy strategies have their limitation, which only can modify the dysfunction of one defective subunit of human complex I. NDI1 protein encoded by yeast *NDI1* gene is a single-subunit protein. It can homologously replace the defective mammalian complex I regardless of which subunit contributes to the impaired function of complex I [15, 16].

The yeast *NDI1* gene is a nuclear gene containing a mitochondrial targeting sequence (MTS). It has been shown that yeast NDI1 expression in rats did not elicit an immune response, possibly because the exogenous protein is located in mitochondria, where it can evade immune surveillance [54]. The effectiveness of yeast *NDI1* gene or its protein product in the treatment of complex I deficiency-related disease models has been demonstrated in several studies [55–57]. In a mouse model of Multiple Slerosis with functionally deficient complex I, the *NDI1* gene was able to rescue axonal damage and neuronal loss, and thus improve visual function [58]. Likewise, in the mouse model of this study, the bilateral gene therapy of NDI1 obviously improved the motor and exploratory behavior. In the *NDI1* treated MPTP-induced PD mice, the morphology and cell survival of neurons in bilateral SNpc were almost maintained at the normal range, accompanied by the decreased inflammatory response in the same region. In addition, the restoration or improvement of mitochondrial oxidative phosphorylation function especially the activity of complex I enzyme were also largely achieved in SN region (Figs. 5, 6, 7, 8 and 9).

Yeast NDI1 is well expressed in MPP^+^/MPTP-induced PD cell culture and mouse models, and it can effectively compensate for the functional defects of mitochondrial complex I, thereby reducing the MPTP-induced injury of dopaminergic neurons in SNpc of mice, with substantial improvements in neuropathologic and neurobehavioral manifestations. Although MPTP is present in the natural environment, paraquat, a MPP^+^ like chemical that can inhibit complex I activity [59, 60] is a widely used herbicide that has been linked to increased PD risk [61, 62]. Our study could provide a research basis for gene therapy of sporadic PD and other diseases with mitochondrial complex I functional defects caused by MPTP like substances.

### Electronic Supplementary Material

Below is the link to the electronic supplementary material.

**Supplementary Figure 1.** NDI1 gene was efficiently expressed and located in mitochondria after transduced into SH-SY5Y cells. A: The GFP (NDI1)-positive cell rate was detected by flow cytometry 120 h post-transduction. B: The HA (NDI1) expression was detected by Western blot. C: Co-localization of mitochondria and HA (NDI1) observed by confocal microscope. The cells were co-stained with red (MitoTracker), green (HA antibody) and blue (DAPI) (scale bar: 25 μm)

## Data Availability

The datasets used and/or analyzed during the current study are available from the corresponding authors on reasonable request.

## Abbreviations

AAV: Adeno-associated virus
rAAV: recombinant adeno-associated virus
FCCP: Carbonyl cyanide 4-(trifluoromethoxy)phenylhydrazone
LCR: Leakage control rate
MPP^+^: N-methyl-4-phenylpyridinium
MPTP: 1-methyl-4-phenyl-1,2,3,6-tetrahydropyridine
NDI1: NADH-quinone oxidoreductase
PD: Parkinson’s disease
RCR: Respiratory control rate
ROS: Reactive oxygen species
SN: Substantia nigra
SNpc: Substantia nigra pars compacta
TH: Tyrosine hydroxylase
VTA: Ventral tegmental area
