# Supplementary figures and images for: Targeting Mitochondrial Complex I Deficiency in MPP+/MPTP-induced Parkinson’s Disease Cell Culture and Mouse Models by Transducing Yeast NDI1 Gene
Source: Biol Proced Online. 2024 Apr 9;26:9. doi: 10.1186/s12575-024-00236-3 (PMC11003148; doi:10.1186/s12575-024-00236-3)

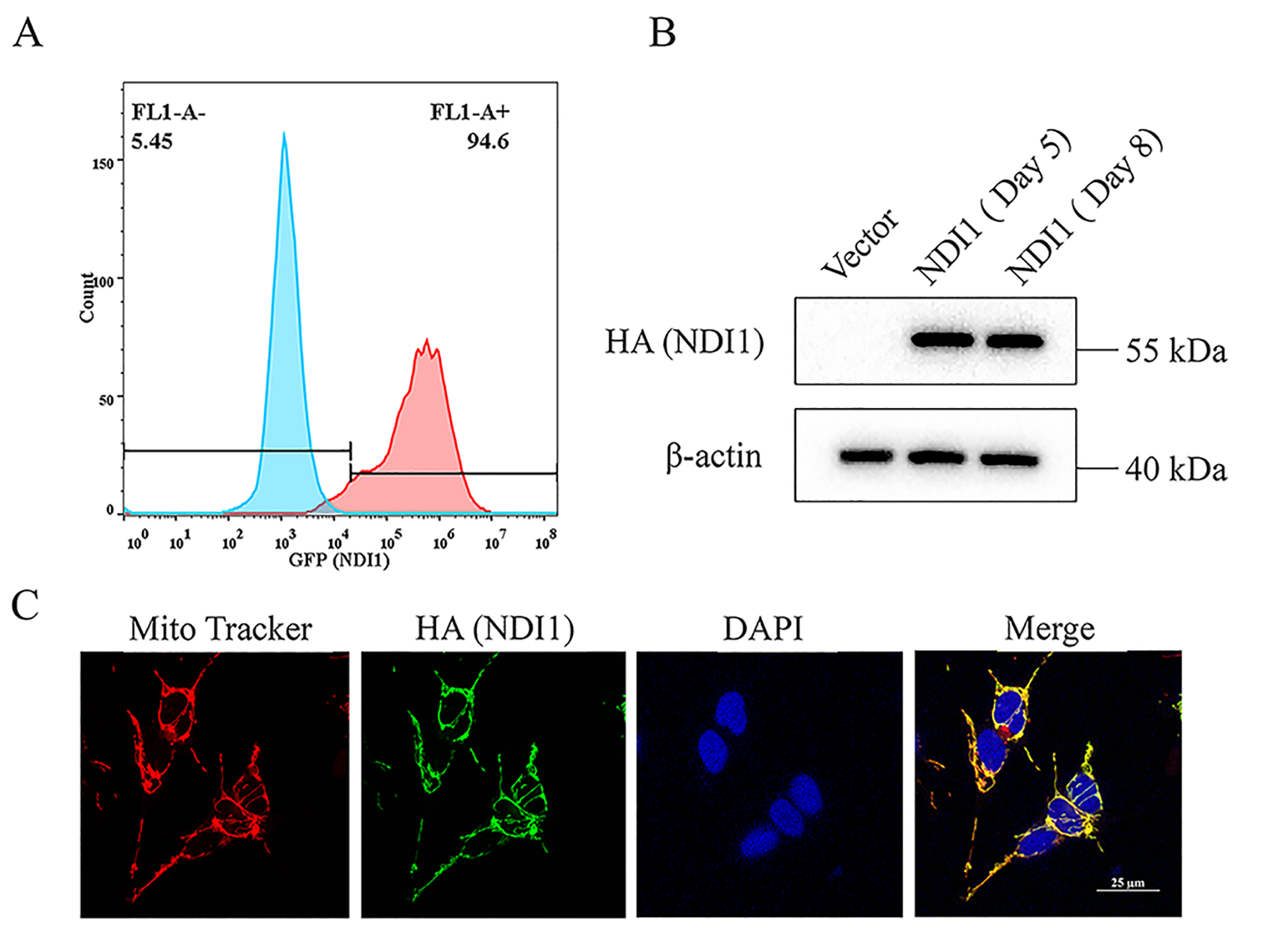

Supplement: Supplementary file 1 — Supplementary Figure 1. NDI1 gene was efficiently expressed and located in mitochondria after transduced into SH-SY5Y cells. A: The GFP (NDI1)-positive cell rate was detected by flow cytometry 120 h post-transduction. B: The HA (NDI1) expression was detected by Western blot. C: Co-localization of mitochondria and HA (NDI1) observed by confocal microscope. The cells were co-stained with red (MitoTracker), green (HA antibody) and blue (DAPI) (scale bar: 25 μm) [file 12575_2024_236_MOESM1_ESM.tif]
